# Leveraging existing program data for routine efficiency measurement in Zambia

**DOI:** 10.12688/gatesopenres.12851.2

**Published:** 2018-11-26

**Authors:** Rick Homan, John Bratt, Gregory Marchand, Henry Kansembe

**Affiliations:** 1 Health Services Research, FHI 360, Durham, North Carolina, 27701, USA; 2 Avencion Ltd., Lusaka, Zambia; 3 Planning Department, Ministry of Health, Government of the Republic of Zambia, Lusaka, Zambia

**Keywords:** efficiency, routine, site-level, Zambia, dashboards, unit expenditure

## Abstract

**Rationale: **As donor contributions for HIV/AIDS stagnate globally, national governments must seek ways to improve use of existing resources through interventions to drive efficiency at the facility level.  But program managers lack routine information on unit expenditures at points of care, and higher-level planners are unable to assess resource use in the health system.  Thus, managers cannot measure current levels of technical efficiency, and are unable to evaluate effectiveness of interventions to increase technical efficiency.

**Phased Implementation of REMS: **FHI 360 developed the Routine Efficiency Monitoring System (REMS)-a relational database leveraging existing budget, expenditure and output data to produce quarterly site-level estimates of unit expenditure per service.  Along with the Government of the Republic of Zambia (GRZ) and implementation partner Avencion, we configured REMS to measure technical efficiency of Ministry of Health resources used to deliver HIV/AIDS services in 326 facilities in 17 high-priority districts in Copperbelt and Central Provinces.  REMS allocation algorithms were developed through facility assessments, and key informant interviews with MoH staff.  Existing IFMIS and DHIS-2 data streams provide recurring flows of expenditure and output data needed to estimate service-specific unit expenditures.  Trained users access REMS output through user-friendly dashboards delivered through a web-based application.

**REMS as a Solution: **District health managers use REMS to identify “outlier” facilities to test performance improvement interventions.  Provincial and national planners are using REMS to seek savings and ensure that resources are directed to geographic and programmatic areas with highest need.  REMS can support reimbursement for social health insurance and provide time-series data on facility-level costs for modeling.

**Conclusions and Next Steps:  **REMS gives managers and planners substantially-improved data on how programs transform resources into services.  The GRZ is seeking funding to expand REMS nationally, covering all major disease areas.  Improved technical efficiency supports the goal of a sustainable HIV/AIDS response.

## Rationale

Global demand for HIV/AIDS services is increasing faster than available resources, resulting in a substantial funding gap. In 2016, a total of US$19.1 billion was spent by all contributors (international and domestic) to the global HIV/AIDS response in low- and middle-income countries (LMICs) ^1^. The UNAIDS “Fast Track” strategy has accelerated the timeline for ending the public health threat from HIV/AIDS, but intensification of effort comes at a high cost: by the year 2020, the global HIV response is projected to require US$26.2 billion in annual resources, a 37% increase over current spending levels ^2^. This gap could grow even larger, due to increases in treatment costs (more people on treatment and more expensive treatment regimens), demand for emergent interventions such as medical male circumcision and pre-exposure prophylaxis and flattening trends in international and domestic HIV/AIDS financing ^3^.

Closing the HIV/AIDS funding gap will require a multifaceted approach, including substantial new resources from international and domestic sources, as well as stronger efforts by national health authorities to increase efficiency of resource use in their respective countries ^4^. But international funding commitments leveled out after the 2009 global financial crisis, and in 2016 worldwide financial pledges declined by 7% over the previous year ^1^. More optimistically, recent analyses of HIV/AIDS service delivery programs suggest substantial scope for cost savings through improvements in technical efficiency. Zeng *et al.* ^5^ noted that existing literature on unit costs of HIV/AIDS interventions shows sizeable variation across programs and countries, much of which is unexplained and likely related to poor governance and weak human resource capacity. The authors conducted an econometric analysis of HIV/AIDS program efficiency in 68 countries and concluded that a typical country program could double its output if it used inputs more efficiently. Remme *et al.* ^6^ analyzed several different policy levers for increasing domestic support for HIV/AIDS programs in 14 sub-Saharan countries and found potential efficiency gains equivalent to 29 percent of current spending. Di Giorgio *et al.* ^7^ examined ART provision in Zambia, Kenya and Uganda and determined that if health facilities boosted their efficiency levels to an 80 percent level, utilization could increase by 33% – 62%.

To realize these potential efficiency gains, managers at all levels of LMIC health systems need information on current technical efficiency in facilities under their supervision. Much of the existing evidence on technical efficiency at the facility level has come from large multi-country cost studies ^8– 10^ or “one-off” studies conducted in single countries ^11– 13^. These studies, typically cross-sectional, have generated useful information on differences in costs across sampled services and facilities within LMIC health systems, across countries and regions, and trends in service costs over time. In 2017 the Global Health Cost Consortium (GHCC) created a “Unit Cost Repository for TB and HIV Prevention, Treatment, and Care Interventions,” (see GHCC Study Repository) which seeks to consolidate unit cost information from HIV/AIDS and TB-related interventions in a searchable database. But few cost studies are nationally-representative, which diminishes their utility in guiding facilities not sampled toward higher technical efficiency. Also, release of study results often lags data collection by one or two years, making it likely that reported findings correspond to a program that has changed substantially in the interim. Finally, these studies themselves can be costly and intrusive to service delivery.

In upper-income countries (UICs), health system managers increasingly are using data-driven cost accounting systems to track and improve performance across networks of similar service outlets. Cost accounting can be differentiated from financial accounting primarily by the intended audience. Cost accounting information is used by decision makers inside the organization to improve cost control and efficiency, while financial accounting information is geared toward external groups such as shareholders, lenders and regulators. Cost accounting systems encompass multiple interoperable databases, and enable managers to monitor patterns of resource use, identify cost drivers for cost control, measure total costs of care at various levels of the health system, integrate information on financial inputs and clinical outputs and outcomes, and improve data utilization through use of executive dashboards (see Becker’s Hospital CFO report on advanced cost accounting). But such systems are expensive to install and maintain, and have not yet been adopted within the LMIC context.

Managers of health systems in LMICs clearly need different data and tools to drive efficiency and effectiveness within facilities under their supervision. The purpose of this Open Letter is to describe an effort – led by the Government of the Republic of Zambia (GRZ) with technical support from FHI 360 and Avencion Ltd. - to develop an automated Routine Efficiency Measurement System (REMS) that uses existing budget, expenditure, and health output data to produce near-real-time estimates of expenditure per unit of service at the individual health facility. REMS incorporates two main innovations: (1) a computational mechanism called a “resource allocation matrix” (RAM) that enables total expenditures at the facility-level to be translated via a set of allocation weights to the program’s main “service lines” and the resources used to support each service line; and (2) a relational database accessed through a web-based portal that combines expenditure data from the existing financial management information system with output data from the DHIS2 system to generate quarterly estimates of expenditure per unit of service at the point of care. Output from REMS can be used by health system managers to monitor expenditure patterns over time, guide resource allocation decisions, and identify best practices for lowering unit costs. The REMS build in Zambia currently covers 326 facilities spread across 17 districts in Central and Copperbelt provinces.

## Phased Implementation of REMS

### Creating a culture of collaboration and government ownership

From the outset, the REMS technical assistance team within FHI 360 and Avencion prioritized formation of a project implementation group led by GRZ officials, reflecting our belief that ownership by the Ministry of Health (MoH), Ministry of Finance (MoF) and Ministry of Community Development and Social Welfare (MCDSW) was critical to the project’s immediate success and long-run sustainability. Support from all three ministries was essential as the Ministry of Finance controls access to the expenditure data, the Ministry of Health manages the health system and resources at the National and Provincial level and the Ministry of Community Development and Social Welfare manages the health system and resources at the District and Community level. We also sought to create a culture where government officials would be empowered to provide active and engaged leadership in project planning and eventual implementation. Besides reflecting our belief in user-centered design, past experience has shown many projects fail when a solution is imposed from outside and then transferred to local operation. This empowerment was accomplished through a concerted effort to engage ministerial senior leadership and, in parallel, by convening regular consultative meetings with integrated teams of government technical experts. Our approach increased investment of time by government senior leadership and subject matter experts. This integrated group of government officials evolved into a REMS core team that championed and guided the project by providing continuous feedback on direction. The core team members were identified through an iterative process of regularly engaging with key leadership within the relevant government ministries to discuss REMS functional requirements, overall implementation, validation and sustainability of the REMS system. REMS core team members were chosen based on their level of commitment, overall interest, and level of influence within the government hierarchy.

At the constitution of the MoH REMs core team, a REMS implementation workshop was held where the core group established team principles, values, and capacity developmental needs which were the basis for the implementation strategy and work plan activities. To establish these principals, values, and capacity developmental needs, the workshop agenda incorporated a team building activity. The results from the core team exercise are shown in Table 1.

**Table 1.** Core group team building result: values, principles, attributes, strengths and capacity developmental needs.

**Core Values and Principles**
Family (spouses and children)
Make sure systems and resources are efficiently utilized
Helping poor and providing access to health services and other human needs
Building Africa’s success story; including our culture and community
Preaching and religious beliefs
Equity and truth
Inclusion and leveraging the African Diaspora
Arts and engineering
**Implementation Team’s Desired Attributes**
Have a winning attitude and pragmatic (making ideas happen)
High performance with a clear focus on targets, objectives and getting things done
Truthful, knowledgeable and with a common understanding
Interoperability and sustainability
Not afraid of change, open minded, innovative and with ability to adapt
Team work/collaboration focused on optimizing talent and expertise
Every team members carries their own weight; accountability and mutual contribution
Supportive, jovial and uplifting
**Implementation Team Strengths**
Problem solving and analysis
Organizing and teaching
Operating on patients (orthopedic surgeon)
Creativity, innovating and finding different ways of doing something
Following up, speak language of equity, analysis
**Implementation Team Areas Requiring Additional Capacity**
Technical writing
Coding / programming (software)
Conflict resolution management
Summarizing ideas and concepts without losing value
Evidence based decision making

### Dissemination of REMS

The REMS project was fully integrated into the sector wide approach (SWAp) mechanism, which is the health sector coordination platform encompassing various layers of technical and policy activities including technical sub-committees, technical working groups (TWGs), policy review meetings and Annual Consultative Meetings (ACMs). TWGs in the Zambian ministerial context report their activities at periodic Policy Meetings composed of senior leadership and Cooperating Partners. By fully integrating the REMS project within the SWAp framework, the core team could regularly disseminate information to sector partners, create linkages with other complementary initiatives in the health ecosystem, build effective working relationships with partners, share user adoption cases and foster continuous support within government. Led by the ministerial core team, the REMS project was continuously represented in the existing SWAp meetings inclusive of Monitoring & Evaluation (M&E) and Healthcare Finance TWG meetings. As a matter of standard practice, REMS presentations at SWAp meetings were always delivered by a Zambian government health official who could speak to the relevance of REMS to supporting health service delivery.

The M&E technical working group in early 2017 met with many health partners at MoH head office where REMS was presented. The feedback from health partners was that a system that can link health outputs and the financial resources mapped to service delivery was long overdue. Since REMS was receiving external data from disparate systems, it also was providing a periodic quality assurance layer for both the financial and health output data. REMS was further presented in the December 2017 Policy Meeting by the MoH’s Department of Policy & Planning and Healthcare Finance teams, culminating in an official endorsement of REMS by the Permanent Secretary of Health as the GRZ’s preferred efficiency measurement tool in the health sector. Thus, by working through existing ministerial structures, REMS became visible at the senior management level and, in parallel, was continuously validated by health sector technical experts, resulting in official acceptance and buy-in.

### Technical description of Zambia REMs build

A key feature and advantage of REMS is that it leverages existing financial and output data, reducing the amount of “new” data needed to generate unit expenditure estimates. Governments routinely produce information on expenditures and outputs, but bringing these two data streams together electronically to produce recurring unit expenditure estimates at the facility level is an innovation. The Zambian MoH uses the Integrated Financial Management Information System (IFMIS) ^14^ to track financial expenditures, and the District Health Information System (DHIS 2) to report on service outputs at the facility level. The Zambia REMS relational database creates an electronic linkage between IFMIS and DHIS2 data in two steps: first by stepping down quarterly IFMIS expenditures (which are often reported at the national, provincial, or district level) to the facility level and allocating expenditures at the facility level to specific services, and second by dividing these allocated expenditures by the number of units of output for that service for the same calendar quarter.

Figure 2 provides a schematic of the logic flow used in the REMS database. In the upper left, the existing IFMIS data system is represented by a green cylinder. Additional data sources required by REMS are the allocation weights shown as the green cylinder in the upper right of Figure 2 and the Facility Resource Allocation Matrices (RAMS) shown in the lower right. These data exist as look-up tables within the REMS database, and were assembled as follows: Allocation weights to isolate HIV-related expenditures and allocate HIV-related expenditures from National level IFMIS accounts to the Provincial or District level were derived from discussions with key MoH informants at national, provincial, and district level. Existing GRZ MoH planning and budgeting documents were then used to develop allocation weights to assign expenditures down to the point of care. These allocation weights were applied to the expenditures reported by IFMIS account number. IFMIS account numbers comprise a unique combination of head/department/unit/programme (and in some cases)/activity numbers. For each IFMIS account controlled at each level, informants were asked to identify what portion of the expenditures in that account were being used to support HIV/AIDS services, and of those expenditures, what portion was flowing down to the lower level(s) vs. remaining at the current account level. Portions of expense accounts that were flagged as HIV/AIDS-related but not flowing down further were classified as “above-facility” expenses.

Once expenditures were tracked to the point of care (facility) the facility RAMS (shown in lower right of Figure 2) were used to distribute HIV-related expenditures incurred at a service delivery point (facility) to specific HIV services delivered in that location (facility or community) and also to classify into expense categories (i.e., labor, drugs, other supplies, equipment, etc.). The facility-specific RAMs were derived from detailed facility assessments in which trained data collectors interviewed clinic staff to determine inputs used to provide specific HIV services within each facility. Data collectors obtained inventories of equipment and supplies used to support service delivery as well as records of HIV-related drugs dispensed in the prior month. This is similar to micro-costing where an ingredients approach is used to build up the cost estimates. Standard unit costs were applied to each resource identified and these data were summarized in an annual prototypical operational budget format for each facility. Each line item in the operational budget was then allocated across the service(s) for which it was used, considering times and locations when services are available, as well as the relative volume of services when resources such as staff or equipment are shared. This budget was then used to compute the percentage of total annual resources used for specific combinations of service and resource type. These percentages were then stored as a RAM weight. Each facility-specific RAM comprises a matrix of weights corresponding to unique combinations of resources and the services they support within that facility. For example, the RAM may tell us that 5% of total annual HIV-related resources are used for personnel providing HTC services. REMS uses this information to assign 5% of each HIV-related Kwacha reaching the facility to personnel for HTC services.

By dividing the allocated expenditures for a specific service at a facility by the volume of service provided during the same time period as reported in the DHIS database (not shown in Figure 2) we were able to estimate the unit cost of service provision at the facility by expense category (shown as blue parallelogram in lower right of Figure 2). This unit cost estimate serves as a performance metric that can be compared across facilities and monitored over time to identify promising service delivery practices for replication in other facilities.

The Zambia REMS system is programmed in Microsoft SQL version 13.0 and the annotated source code is available through Zenodo ^15^. REMS uses C# for the user interface in which users select parameters of interest (period, organization, geographic level, service(s) of interest, type of resource(s), facility(s) and total or unit cost. The results of these selections are displayed graphically using HighChart to generate visualizations and REMS allows the user to save the most relevant performance metrics to an individual dashboard (see animation of REMS dashboard from REMS site).

**Figure 1.**
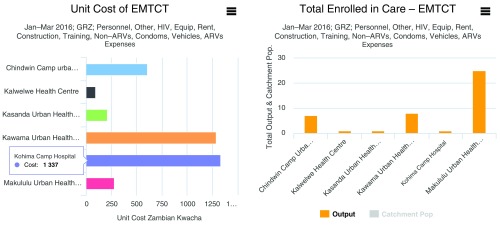
Variability in unit cost and output for EMTCT Services, selected facilities in Central Province.

In the screen shot below ( Figure 1), user-generated results show the unit cost of Elimination of Mother to Child Transmission (EMTCT) services for 6 facilities for the period January–March 2016. In the left panel, a ten-fold variation is apparent in the unit costs of an EMTCT visit (range: <125 Kwacha (~$13) in Kalwelwe Health Centre to 1,337 Kwacha in Kohima Camp Hospital (~$134)). In the right panel, the volume of services (patient visits for EMTCT) is shown for the same time period and facilities. While we would expect that facilities with larger service volumes would have lower unit costs (as they can spread fixed costs across more visits) some noticeable exceptions exist: Kawama Urban Health Centre reports high volume and high unit costs, while both Kalwelwe Health Centre and Kasanda urban Health Centre exhibit low volumes and low unit costs. REMS users at the District Medical Office are trained to analyze comparative unit expenditures across a range of facilities in their district, and also to track trends in unit expenditure over time; thus, both exceptions would serve as prompts for further investigation by the District Health Management team. These results can be viewed as a sliced bar or stacked column that would show the type of resource contributing to the results (i.e., staffing, supplies, equipment, etc.). This detail allows for a comparison of how productive the fixed resources are being utilized as these are what largely drive any potential efficiency gains.

**Figure 2.**
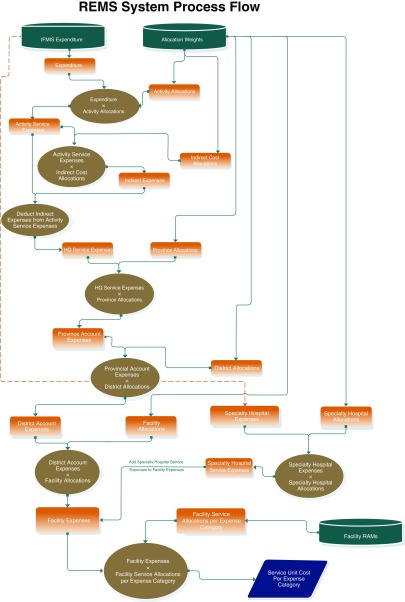
REMS system process flow.

Users at a Provincial Management Office (PMO) have broader permissions to monitor performance and resource efficiency in select priority districts or facilities. At the provincial and national level, users can examine whether overall expenditures are being directed to services or geographic areas where needs are greatest, and can monitor trends in service-level versus above-facility expenditure. By leveraging existing data systems and flows, REMS capitalizes on prior investments in health management information systems and increases the value of the data for decision making.

## REMS as a Solution

In the current environment of scarce resources and growing demand for health services, there exists an urgent need to ensure more efficient use of *existing* resources. An ongoing problem in LMIC health systems is lack of data to measure and monitor economic performance at the service delivery level. Information on technical efficiency from cost studies is not sufficient for day-to-day program management that seeks to drive efficiency and effectiveness in front-line health facilities. As a business intelligence platform, REMS delivers routine information on unit expenditure by type of service, type of expense, time period and funding source, covering all facilities within a health system. We envision several use cases for LMIC managers and decision makers using REMS information, including the following:

• Performance improvement – REMS is enabling Zambian officials at district and provincial levels to carry out routine monitoring of technical efficiency. For the first time, differences in high and low-performing facilities and districts are visible, allowing managers to develop and monitor initiatives that translate into improved efficiency outcomes. Focusing on “outliers” – i.e., facilities or districts with high or low expenditure per unit of output relative to a standard – managers can investigate potential reasons for the differences. For example, differences in program output may be driving unit expenditure variation in similar facilities. If so, what are the underlying reasons for differences in output and how might these be addressed? Another driver of unit expenditure could be staffing levels that are not aligned with patterns of demand in a facility, opening the possibility of reassigning excess staff to busier facilities. Prior to REMS these differences were obscured, making it impossible to identify efficiency differences, or to measure the impact of changes designed to improve efficiency.
• Resource allocation at provincial and national levels - Planners in higher levels of the health system can use REMS expenditure roll-ups by district and province to analyze resource flows, ensuring that funding and services are directed to geographic areas where disease burden is greatest.
• Assessment of HIV/AIDS financing and sustainability – National-level health officials now have information on total expenditure per unit of service, and when the system is fully operational, how the expenditure burden is distributed across different funding entities such as the Global Fund and PEPFAR. They also will be able to document facility and above-facility-level expenditure, and examine trends in the GRZ share of the HIV/AIDS financing burden.
• Support implementation of social health insurance – the GRZ is assessing feasibility of a Social Health Insurance Scheme but lacks a source of routine information on expenditure per unit of service. REMs generates time-series data on unit expenditures that will enable calculation of benchmark unit expenditures that can be used to set reimbursement rates. REMs data also may help planners decide on the design of the system (i.e., claims versus capitation), and could assist in measuring the administrative burden associated with claims.

Recent experience with REMS users in Copperbelt and Central provinces confirm that REMS data are opening doors to new insights into the economics of service delivery. Users are working together to explore some of the “whys” behind indicators in the REMs dashboard. For example, at User Conferences in both provinces in late 2017, mixed groups comprising MoH planners and accountants from District and Provincial levels were tasked with building REMS charts based on specific scenarios. Facilitator-led discussions assisted users to analyze differences in unit expenditure across service delivery points and over time, focusing on potential causes of variation displayed in the charts. User familiarity with health facilities enabled informed discussions about such factors as differential staffing patterns, procurement of vehicles and drugs, and variability in catchment population leading to disparate levels of output. In certain cases, users could cite no obvious reasons for differences in unit expenditures, highlighting a need for further investigation at the facilities themselves ^16^.

In addition to these direct applications of REMS data, we foresee additional indirect benefits that contribute to health system strengthening in various ways. First, existence of routine comparative data on efficiency will engage local staff in efficiency improvement, incentivizing them toward taking a more active role in interpreting and acting on their own data. REMS data also enable supervisory staff to prioritize their support visits to facilities where the need is greatest, and to evaluate whether their support is having the desired effect over time. Second, any sudden or unexpected changes in REMS unit expenditures will trigger scrutiny, and the quality of data streams feeding REMS will be considered as a possible cause of any changes. As an example, while DHIS 2 data are collected at facility level, they are subsequently rolled-up to district and provincial levels for ordinary reporting purposes. If DHIS 2 data are keyed incorrectly or are missing at the facility level, the roll-up may obscure these errors. But REMS uses *facility-level* DHIS2 data as denominators for unit expenditure estimates, and so data entry errors (or omissions) will be immediately obvious in the district-level REMS dashboard screen that compares unit expenditures across facilities. Similarly, any data quality issues in the IFMIS also should be reflected in the REMs dashboard. Thus, increasing reliance on REMS data will focus greater attention on the quality of data that feed the system. Third, access to detailed budget information at the facility level is uncommon in Zambia and most other LMICs. Detailed facility budgets produced by the REMS facility assessment tool will provide local managers with additional information to improve program implementation and increase transparency.

In the current Zambia REMS build, health planners and managers are using the system to monitor efficiency of HIV/AIDS service delivery. REMS was designed purposely to be adaptable for use in other areas such as malaria, child nutrition, and immunization, as well as other global development sectors outside of health, such as education, nutrition and economic livelihoods. REMs requires a set of conditions to be in place before the system can be implemented. Data requirements include budgets at the service delivery level and above; an electronic financial management system like IFMIS; and use of DHIS-2 or a similar electronic application for tabulating system outputs. These conditions already are in place in many LMICS and across multiple development sectors, creating multiple opportunities to bring routine efficiency measurement and monitoring to other sectors and countries.

In summary, the introduction of REMS in two provinces of Zambia addresses the problem of insufficient information on economic performance of healthcare delivery at the site level. REMS produces near-real-time data on expenditure per unit of service, emulating business intelligence typically produced by enterprise resource planning (ERP) software. In Copperbelt and Central provinces, managers now can compare current unit expenditures across facilities and districts, highlighting those sites that are producing services at high or low relative levels of efficiency. We think of REMS as analogous to a thermometer. It can be used to document a fever, but it does not tell the user how to treat the fever. The user is presented with REMS output to help target investigations of facility performance, identify outliers, or perhaps identify data quality issues within the IFMIS or DHIS2 data. If corrective action is taken, REMS (like a thermometer) can be used to assess whether the patient is responding to the treatment and if not lead to further investigations. Our goal is to put actionable information into the hands of persons who manage health systems. This information currently does not exist. Asking health systems to become more efficient users of scarce resources, while providing no means of monitoring resource use is doomed to failure. All too often we see a standard resource complement assigned to a facility based solely on its designation in the health system hierarchy and not tied to the service volumes for which it is expected to deliver.

For the first time in a LMIC, routine data are available to support economic changes to enable the health system to serve more HIV/AIDS clients with the same resources, reducing the fiscal gap currently facing many countries struggling to control HIV/AIDS epidemics. REMS also can be used to monitor the impact on efficiency of innovations such as task-shifting, which a recent literature review of HIV/AIDS and TB programs suggests can lead to efficiency improvements and cost savings ^17^. In addition to the facility-level performance improvements mentioned above, REMS also can be used to estimate above-facility expenditures, which, if managed effectively, may create opportunities to expand service availability within the current resource envelope.

## Next steps

While our award was funded to 1) develop a prototype, 2) secure participation and commitment from the Government of Zambia, and 3) implement the system in two provinces, we are seeking funding to take the system to the next level (expand geographically and programmatically), introduce additional feedback loops within the program logic, bring in additional data systems such as HMIS as it matures, and create a user-interface to generate routine performance reports and query the facility assessment data. In addition, we will work with the MoH to document changes in resource deployment resulting from REMS insights, and measure the impact of these changes on unit cost of services. This validation of REMS, demonstrating how use of the system leads to program improvement, will be essential to the further development of REMS and potential adoption in other countries. We are not claiming to have produced a perfect performance monitoring system but rather to have laid the foundation stones for a more complete and robust system. In an era where countries are being asked to take on a larger share of the financing of the health sector, tools to enable effective management of health systems are necessary.

## Data availability

The source databases used in the development of REMS include DHIS2, IFMIS, and results of the facility assessments conducted for this project (see Table 2). DHIS2 is a system being used to capture statistical data on health activities throughout the country. IFMIS provides budget and expenditure data for government ministries. The facility assessments give details of inputs, service delivery patterns and resource use for each facility. In the scale-up phase of REMS, we expect that facility assessments will become routine annual exercises to document inputs and resource use at MoH facilities, and to form the basis for activity budgets. Below is a summary table of the various databases from which REMS draws its data inputs:

**Table 2.** List of data repositories used in this study.

Repository	Dataset	URL
**DHIS2**	**Health Outputs**	**www.zambiahmis.org**
**IFMIS**	**Financial Inputs**	**Private closed network**
**REMS** **Database**	**Resource** **Allocations**	**Private closed network**

## Access to private closed networks

The full financial / general ledger data extract from the Government of the Republic of Zambia Ministry of Finance IFMIS (Integrated Financial Management Information System) is available to authorized users from the Zambian government and approved partners. Access to back-end system data in IFMIS and the data extract is accessible with written approval from the Ministry of Health Permanent Secretary. For full data availability protocol please contact andrew.kashoka@moh.gov.zm.

The REMS database is hosted on a server at the Ministry of Health in Lusaka, Zambia. User access is limited to approved users from the Ministry of Health, other relevant government agencies and approved partners. Users with full-privileges can access back-end system data and can extract excel and CVS files of resource allocations and expenditures per facility / health service. For full data availability protocol please contact andrew.kashoka@moh.gov.zm.

## Software availability

The Zambia REMS annotated source code is available from GitHub: https://github.com/rhoman88/REMS/tree/v1.0

Archived source code at time of publication: https://doi.org/10.5281/zenodo.1341782 ^15^

License: Apache License 2.0.

## References

[1] Joint United Nations Programme on HIV/AIDS (UNAIDS) and The Henry J Kaiser Family Foundation: Donor Government Funding for HIV in Low- and Middle-Income Countries in 2016. Menlo Park and Washington DC;2017 Reference Source

[2] Joint United Nations Programme on HIV/AIDS (UNAIDS): Fast-track update on investments needed in the AIDS response. Geneva: UNAIDS;2016 Reference Source

[3] Piot P Abdool Karim SS Hecht R: Defeating AIDS--advancing global health. *Lancet.* 2015;386(9989):171–218. 10.1016/S0140-6736(15)60658-4 26117719

[4] Vassall A Remme M Watts C: Financing essential HIV services: a new economic agenda. *PLoS Med.* 2013;10(12):e1001567. 10.1371/journal.pmed.1001567 24358028 PMC3866083

[5] Zeng W Shepard DS Chilingerian J: How much can we gain from improved efficiency? An examination of performance of national HIV/AIDS programs and its determinants in low- and middle-income countries. *BMC Health Serv Res.* 2012;12:74. 10.1186/1472-6963-12-74 22443135 PMC3353196

[6] Remme M Siapka M Sterck O: Financing the HIV response in sub-Saharan Africa from domestic sources: Moving beyond a normative approach. *Soc Sci Med.* 2016;169:66–76. 10.1016/j.socscimed.2016.09.027 27693973

[7] Di Giorgio L Moses MW Fullman N: The potential to expand antiretroviral therapy by improving health facility efficiency: evidence from Kenya, Uganda, and Zambia. *BMC Med.* 2016;14(1):108. 10.1186/s12916-016-0653-z 27439621 PMC4952151

[8] Marseille E Dandona L Marshall N: HIV prevention costs and program scale: data from the PANCEA project in five low and middle-income countries. *BMC Health Serv Res.* 2007;7:108. 10.1186/1472-6963-7-108 17626616 PMC1936993

[9] Galárraga O Wamai RG Sosa-Rubí SG: HIV prevention costs and their predictors: evidence from the ORPHEA Project in Kenya. *Health Policy Plan.* 2017;32(10):1407–1416. 10.1093/heapol/czx121 29029086 PMC5886164

[10] Tagar E Sundaram M Condliffe K: Multi-country analysis of treatment costs for HIV/AIDS (MATCH): facility-level ART unit cost analysis in Ethiopia, Malawi, Rwanda, South Africa and Zambia. *PLoS One.* 2014;9(11):e108304. 10.1371/journal.pone.0108304 25389777 PMC4229087

[11] Obure CD Vassall A Michaels C: Optimising the cost and delivery of HIV counselling and testing services in Kenya and Swaziland. *Sex Transm Infect.* 2012;88(7):498–503. 10.1136/sextrans-2012-050544 22859498 PMC3595498

[12] Aliyu HB Chuku NN Kola-Jebutu A: What is the cost of providing outpatient HIV counseling and testing and antiretroviral therapy services in selected public health facilities in Nigeria? *J Acquir Immune Defic Syndr.* 2012;61(2):221–225. 10.1097/QAI.0b013e3182683b04 22820805

[13] Bratt JH Torpey K Kabaso M: Costs of HIV/AIDS outpatient services delivered through Zambian public health facilities. *Trop Med Int Health.* 2011;16(1):110–118. 10.1111/j.1365-3156.2010.02640.x 20958891

[14] Rodin-Brown E: Integrated financial management information systems: a practical guide. The Louis Berger Group, Inc. and Development Alternatives, Inc. under the Fiscal Reform and Economic Governance Task Order, GEG-I-00-04-00001-00 Task Order No. 06.2008 Reference Source

[15] rhoman88: rhoman88/REMS: REMS Zambia (Version v1.0). *Zenodo.* 2018 10.5281/zenodo.1340656

[16] Chrispin C: Personal communication.2018.

[17] Seidman G Atun R: Does task shifting yield cost savings and improve efficiency for health systems? A systematic review of evidence from low-income and middle-income countries. *Hum Resour Health.* 2017;15(1):29. 10.1186/s12960-017-0200-9 28407810 PMC5390445

