# Supplementary material for: Leveraging existing program data for routine efficiency measurement in Zambia
Source: Gates Open Res. 2018 Nov 26;2:40. Originally published 2018 Aug 31. [Version 2] doi: 10.12688/gatesopenres.12851.2 (PMC6480548; doi:10.12688/gatesopenres.12851.2)
Supplement: Peer review history

# Peer review: Reviewer response for version 2

**DOI:** 10.21956/gatesopenres.13978.r26857

**Published:** 2019-02-19

**Authors:** Tyler Smith

**Affiliations:** 1 Cooper/Smith, Washington, DC, USA

I commend the authors on drafting a timely Open Letter that thoroughly explains a complicated subject matter, clearly articulates a pressing challenge, and proposes a solution demonstrated at country level. 

 The sub-Saharan Africa demographic ‘bulge’ matched with flat or marginal increases in HIV/AIDS funding will likely hamper efforts to achieve the UNAIDS 2030 Fast Track targets. As Dr. Anthony Fauci stated on many occasions, we have the programmatic tools at our disposal, but perennially face an ‘implementation gap’ that prevents us from adequately and efficiently deploying our collective resources and achieving the scale and coverage necessary to ending the epidemic. As the authors assert, measuring efficiency is increasingly paramount.  

 The authors are also correct that decision-making is often hamstrung by a dearth of quality, fit-for-purpose resource data, both for those available in the future and those already deployed. Country governments are routinely asked for data-driven concept notes, investment cases, and funding proposals that include resource projections and plans for stronger accountability, efficiency gains, and measured increases in domestic resource contribution. However, systems to institutionalize access to these data – including validation/quality assurance – remain elusive. 

 Further, capturing the full spend towards achieving a particular program target at facility level is not easy. It requires, not only sophisticated methods and digital systems, but also extensive political and technical negotiations with those that are closest to the point of service and cost centers. The authors did a great job of outlining the necessity of country ownership and the steps taken for stakeholder consultation/feedback. These both go a long way to demystify the math and garner critical buy in of the results for use in decision-making. 

 In general, the REMS platform and approach may help to rectify a glaring monitoring gap and provide easy to use tools for better program management, but there are some notable caveats:

  

1. For unit costs to be useful, all sources of funding must be included: 
  As illustrated in Figure 2, it appears that the only financial data used in REMS comes from the Government’s financial management system - IFMIS. It would be useful for the authors to clarify if REMS includes only domestic resources or if it is inclusive of the other major HIV funding streams namely PEPFAR and Global Fund. Given that external sources often make up the bulk of HIV program resources, it’s critical that these are included in any estimates of unit cost. If these data are not currently captured, how would they be in the future using REMS? Partners have traditionally been hesitant to release the full extent of the financial data they gather, limiting country efforts toward a unified financial monitoring system for health and HIV. Has this been a challenge in the Zambian context and how would it be handled in other use cases? 
2. Transparency and easy adjustment to allocation coefficients must be the norm:  
  Though estimating unit inputs for HIV care and treatment at each facility sounds rigorous, this approach may yield varied results based on the quality of the enumerators and the understanding of health workers at the time of data collection. There may not be a better method at current, but it would be interesting if the authors could comment on the degree to which the RAMS coefficients vary and if clear patterns emerge – i.e. different intensity based on size of HIV patient cohort, size of facility, external technical assistance, etc. Further, is there a process in place for how the RAMS are accessed, updated, and validated routinely? We hope HIV services evolve as new and more efficient means of HIV treatment are incorporated, so the coefficients assigned at facilities will have to keep up to remain accurate and relevant. 
3. The results must be validated in context:
  Given the REMS platform represents a new approach to allocating expenditures and generating unit costs, it is important the results are triangulated with other methods and data sources and the disparities are highlighted for further investigation. Have unit cost results thus far been compared with related sources – e.g. the PEPFAR unit expenditures for similar indicators and/or more traditional cost studies? Are they similar or dramatically different? Though variation is expected for a number of reasons, a deeper triangulation will help to understand how best to interpret the results and how they should be applied in planning and management.
4. Progress to a global good:
  It’s clear the authors are keen to expand the REMS system and deploy it to additional sites, countries, health programs, and sectors. A major challenge for health programs currently is the emergence of siloed data streams and digital systems, resulting in fragmentation and incongruence of data that limits application and value. Many countries have developed eHealth national strategies and articulated a vision for an enterprise system architecture that is efficient, streamlined, and government owned. Given the inherent value of a system like REMS, matched with its broad applicability, perhaps the authors would consider positioning the platform as a global digital good – an open-source solution that is designed to plug and play nicely with other country level systems (e.g. DHIS2). Preparing this product for an open source deployment would require harmonization with global standards for interoperability and substantial, ongoing technical support to operationalize the platform at country level. 

 

 Finally, the authors provide good example use cases of how the routine outputs of REMS could be used, but it would be helpful to outline concrete examples of how the data have been used in Zambian health facilities to adjust resource allocations, improve service delivery, or trigger deeper investigations. 

 In sum, the REMS platform provides a promising solution to fill a critical data gap and could be widely deployed if the financial data captured are comprehensive (including non-government resources), the allocation methods are transparent and easily adjusted/updated, the results are validated/triangulated, and the platform evolves to a public good that easily integrates with other systems in an enterprise fashion.

I have read this submission. I believe that I have an appropriate level of expertise to confirm that it is of an acceptable scientific standard.

# Peer review: Reviewer response for version 2

**DOI:** 10.21956/gatesopenres.13978.r26850

**Published:** 2019-02-04

**Authors:** Donald S. Shepard

**Affiliations:** 1 Schneider Institutes for Health Policy, Heller School, Brandeis University, Waltham, MA, USA; 2 Carnegie Mellon University in Australia, Adelaide, South Australia, Australia; 3 Flinders University, Adelaide, South Australia, Australia

This nicely written Open Letter describes the creation and illustrative output from the Routine Efficiency Monitoring System (REMS). The salient idea of REMS is linking data on inputs from the country’s financial system maintained by its Ministry of Finance with its output system (numbers of health services delivered) at the facility level by quarter. This linkage and the associated dashboard allows managers to determine and visualize unit costs by service, facility or district, and by quarter. The authors note that the system has been piloted in Zambia’s Central and Copperbelt Provinces. The strength of this system is best summarized in following excerpt:

 "We think of REMS as analogous to a thermometer. It can be used to document a fever, but it does not tell the user how to treat the fever. The user is presented with REMS output to help target investigations of facility performance, identify outliers, or perhaps identify data quality issues within the IFMIS or DHIS2 data. If corrective action is taken, REMS (like a thermometer) can be used to assess whether the patient is responding to the treatment and if not lead to further investigations."

 The authors describe a participatory system across government agencies and partners intended to build a sustainable system. It would informative to read about how long the system has been in operation and how sustainable the model has proved.

 As donors and governments decide on the value of maintaining and possibly extending such a system, several pieces of information would be useful. Following their fever analogy, it would be helpful to understand the incidence of “outliers” that they uncovered. How often were the outliers related to omissions or mistakes in financial data or service utilization data? When data issues were identified, were they one-off or systematic? Where the outliers were related to facility performance, the authors noted that smaller facilities would be expected to have higher costs, as they had less volume against which to distribute the fixed costs. It would be helpful to develop volume-adjusted standardized costs that recognize these fixed costs. Then actual unit costs could be compared against these standards for a more meaningful comparison.

 Facilities with high costs could be candidates for reallocating personnel or supplies; ones with low costs might illustrate exceptional good practices, or reflect understaffed facilities trying to cope with high demand.

 The authors did not describe the cost in developing REMS. While they noted that they were seeking funding for possible extensions, they did not describe their projections. Policy makers would be interested both in extending the system to additional provinces in Zambia as well as to another country with parallels in data systems. Both of these financial items would be informative.

 Policy makers are often familiar with the 80-20 rule, where 80 percent of the value could be obtained with 20 percent of effort. Applying that rule in this case, policy makers may wish to consider two simplified versions.

 One simplification would relate to the number of services reported. If the “Resource Allocation Matrix” uses a set weight for each type of service (which is a plausible process in view of the available data), then a facility with a cost of three times the mean for one service will have three times the mean for every other service. In that case, the unit costs could simply be reported for one benchmark service, such as a “standard facility visit.” Each type of service would have a weight relative to a standard visit.

 A similar process determined the cost per outpatient visit and cost per bed day from a hospital visit (Shepard *et al.*, 2000 ^1^). A district manager could examine the cost per standard visit across the health centers in his/her district in a given quarter, as well as the evolution of these unit costs over time in a single health center. A similar process could examine costs per bed day across hospitals.

 The second simplification relates to the level of the delivery system. Instead of analyzing data at the facility level, it may be informative to examine allocations at the district level. This aggregation would cut the number of units by an order of magnitude. Supplies and personnel are often allocated to districts, so the quality of data and ability to act may be far greater.

I have read this submission. I believe that I have an appropriate level of expertise to confirm that it is of an acceptable scientific standard, however I have significant reservations, as outlined above.

## References

[1] ShepardDSHodgkinDAnthonyY: Analysis of hospital costs : a manual for managers / Donald S. Shepard, Dominic Hodgkin, Yvonne Anthony. *World Health Organization*.2000; Reference source

# Peer review: Reviewer response for version 2

**DOI:** 10.21956/gatesopenres.13978.r26778

**Published:** 2019-01-14

**Authors:** Stefano Michele Bertozzi, Madhav Nekkar

**Affiliations:** 1 School of Public Health, University of California, Berkeley, Berkeley, CA, USA; 2 University of California, Berkeley, Berkeley, CA, USA

The authors have responded well to the concerns expressed in the initial review and have reclassified the submission as an "open letter" rather than a "research article".  In light of these changes, I am pleased to change my approval status to "approved". Thank you for having invited us to review.

We have read this submission. We believe that we have an appropriate level of expertise to confirm that it is of an acceptable scientific standard.

# Peer review: Reviewer response for version 1

**DOI:** 10.21956/gatesopenres.13932.r26632

**Published:** 2018-10-04

**Authors:** Stefano Michele Bertozzi

**Affiliations:** 1 School of Public Health, University of California, Berkeley, Berkeley, CA, USA

Overall, the paper is well-written and highlights a very important issue (the need for better data collecting and aggregation) that is worth addressing. The paper is a description of the development and implementation by FHI 360 of a version of a health management information system they call REMS, or the Routine Efficiency Measurement System. The system takes the financial data from the Integrated Financial Management System and service delivery data from the DHIS2 system and uses those data to generate unit-specific efficiency estimates. The authors describe the system and its successful implementation in partnership with the MoH of Zambia. The paper addresses the process that was followed with the Government of Zambia to shape the REMS and ensure that it is responsive to the GoZ’s needs.  It also describes the implementation of the system, the characteristics of the system, and some preliminary evidence of the system’s utility to its users. 

 Concerns:

- The paper shoehorns a description of the implementation of a health management information system into a background/methods/results/conclusion format as if this were a research study addressing a research question. This is not a research paper, but rather a well written journalistic recounting of a project designed to improve the collection and analysis of efficiency in Zambia. It doesn’t have a discernible research question; it doesn’t apply discernible research methods to answering a research question.
- The paper claims that:
*As a business intelligence platform, REMS delivers routine information on unit expenditure by type of service, type of expense, time period and funding source, covering all facilities within a health system.* 
  Implied in the claim is that the REMS system is delivering accurate, useful, actionable data about performance of clinics within the system. It estimates unit expenditure by type of service using Facility Resource Allocation Matrices (RAMS) to allocate facility-level costs among the different activities in facilities. The authors mention that the “allocation weights are based on input from key MoH informants” and that the Facility Resource Allocation Matrices (RAM) “weights were derived from a detailed facility assessment” with interviews but that is the extent of the description about how exactly the matrix determines unit cost. However there is no reported validation of this approach. The reader has no reason to have confidence that the estimates generated by the system are sufficiently accurate so as to be actionable. There is only the implicit suggestion that because the Ministry has started to use the system to assess efficiency therefore the data generated must be useful. While I am a big believer in the power of data that reveals heterogeneity in performance to improve performance across the system, it too much to expect blind trust in a new method to estimate efficiency.  How much of the estimated heterogeneity (variation in efficiency among clinics) is real and how much is an artifact introduced by the estimation tool?
- The paper implicitly makes the claim that efficiency data are very important for improving system performance (with which I agree, as a general point) and that the REMS system, in providing such data, is thus transforming health system efficiency. While this story is plausible, no evidence is presented that the interest expressed by the MoH in the REMS data are actually leading to transformational changes in health system performance. For example, the paper offers graphs of unit costs of certain types of care across different healthcare facilities showing highly heterogeneous costs per unit of service delivered. However, there is no indication of whether the large differences in cost of EMTCT are just a reflection of differences in maternal HIV prevalence – or whether there are clear differences in the efficiency of the delivery of the services provided. Thus, it is not clear from the paper that the estimation approach used will meaningfully result in better allocation of resources and improve efficiency.
- The paper makes several statements that argue that the process followed by FHI 360 was critically important to the success of the implementation. One such example:
   
*Ensuring ownership by the Ministry of Health (MoH), Ministry of Finance (MoF) and Ministry of Community Development and Social Welfare (MCDSW) was critical to the project’s immediate success and long-run sustainability.*
  All of these statements are plausible, but there is no evidence provided to support the assertions. Would the implementation of a similar system in another country require the same degree of complexity in the implementation process? Is there evidence that the implementation would not have been successful if it had just been implemented by the MoH and subsequently revealed to the other Ministries? etc. As such, they appear to be statements of opinion by the authors that the approach they took was the correct/efficient/ethical approach that should be replicated if another country were to consider implementing such a system. It perhaps goes without saying that it is especially important to qualify such opinions when the authors are those who implemented the approach being assessed. A scientific article would normally make such statements only after having tested and validated the assertions using some scientific method and using observers who are as unbiased as possible.      

Thus, the paper in its current form is not a scientific publication but rather a well-written, well-organized report of the development and implementation of a health data system. It should not be published in the form of a scientific publication but rather as a report. Even as a report it should acknowledge (if true) the lack of rigorous validation of its estimation method and call for such validation. Such validation would not only give the Government of Zambia greater confidence in the estimates the system produces but provide much more useful information to other countries considering implementation of a similar system.

 In addition, the report should recognize that comparisons of unit costs without any assessment of the sources of the unit cost differences can provide seriously misleading information to decision-makers. If they are going to share examples of revealed heterogeneity being usefully addressed, it would be very helpful to show that the system can identify the source of the cost differences and that those have led to changes that have improved system performance. I often refer to justifiable and unjustifiable heterogeneity (which depends upon the perspective of the observer). As an example, within Zambia there is more than an order of magnitude of difference in HIV prevalence from one locality to another. It stands to reason that the unit cost of HIV services (especially prevention services) would therefore also (justifiably) vary greatly from one locality to another. Revealing (true) heterogeneity among localities can therefore represent differences in efficiency of service delivery (that needs to be addressed with improved management of service delivery) or differences in return to investment in prevention (which might be addressed by reallocating prevention services to localities where the returns are greater). At a minimum the report should temper the enthusiasm of the authors regarding the utility of the raw system output – or it should reveal that the system output is already far more nuanced than the current example suggests.

 I would like to acknowledge the assistance of Madhav Nekkar in preparing this review. I accept full responsibility for its content.

I have read this submission. I believe that I have an appropriate level of expertise to state that I do not consider it to be of an acceptable scientific standard, for reasons outlined above.

# Response

**Published:** 2018-11-01

**Authors:** John Bratt

Overall, the paper is well-written and highlights a very important issue (the need for better data collecting and aggregation) that is worth addressing. The paper is a description of the development and implementation by FHI 360 of a version of a health management information system they call REMS, or the Routine Efficiency Measurement System. The system takes the financial data from the Integrated Financial Management System and service delivery data from the DHIS2 system and uses those data to generate unit-specific efficiency estimates. The authors describe the system and its successful implementation in partnership with the MoH of Zambia. The paper addresses the process that was followed with the Government of Zambia to shape the REMS and ensure that it is responsive to the GoZ’s needs.  It also describes the implementation of the system, the characteristics of the system, and some preliminary evidence of the system’s utility to its users. 

Concerns:

- The paper shoehorns a description of the implementation of a health management information system into a background/methods/results/conclusion format as if this were a research study addressing a research question. This is not a research paper, but rather a well written journalistic recounting of a project designed to improve the collection and analysis of efficiency in Zambia. It doesn’t have a discernible research question; it doesn’t apply discernible research methods to answering a research question.

 
**Response from the Gates Open Research Editorial Office** **: *The article by Homan et al was originally classified as a Research article by the Gates Open Research Editorial Office due to the presence of methodology and software source code. However, on a re-assessment of the article, and taking the comments of reviewer 1 into consideration, we would suggest that this article should be reclassified as an **Open Letter*** ***. Open Letters discuss policies relevant to a broad research community, present guidelines or announce new initiatives. The article by Homan et al describes the development and implementation of a new initiative, thereby fitting the criteria for an Open Letter*** *.*  

- The paper claims that: *As a business intelligence platform, REMS delivers routine information on unit expenditure by type of service, type of expense, time period and funding source, covering all facilities within a health system.* Implied in the claim is that the REMS system is delivering accurate, useful, actionable data about performance of clinics within the system. It estimates unit expenditure by type of service using Facility Resource Allocation Matrices (RAMS) to allocate facility-level costs among the different activities in facilities. The authors mention that the “allocation weights are based on input from key MoH informants” and that the Facility Resource Allocation Matrices (RAM) “weights were derived from a detailed facility assessment” with interviews but that is the extent of the description about how exactly the matrix determines unit cost. However there is no reported validation of this approach. The reader has no reason to have confidence that the estimates generated by the system are sufficiently accurate so as to be actionable. There is only the implicit suggestion that because the Ministry has started to use the system to assess efficiency therefore the data generated must be useful. While I am a big believer in the power of data that reveals heterogeneity in performance to improve performance across the system, it too much to expect blind trust in a new method to estimate efficiency.  How much of the estimated heterogeneity (variation in efficiency among clinics) is real and how much is an artifact introduced by the estimation tool?

**Response from the Authors** ***: There is a lack of clarity here that will be addressed in the revised manuscript.  The allocation weights based upon key MoH informants are the weights used to: 1) isolate HIV-related expenditures within the IFMIS accounts and 2) allocate HIV-related expenditures from National level IFMIS accounts to the Provincial or District Level.  Key MoH informants also provided information to isolate HIV -related expenditures in IFMIS accounts pertaining to Provincial or District Level, but then existing GRZ MoH planning and budgeting data were used to further allocate these accounts down to the point of care.  Once expenditures are tracked to the point of care, the facility-specific RAM is used to allocate those expenses to specific services and resource categories.  The facility-specific RAM is derived from facility assessments where facility staff identified the resources used to support each HIV service within the facility.  This is a similar process used in micro-costing, where an ingredients approach is used to build up the cost estimates.  The facility-specific RAM is constructed in two steps: first, by creating an annual operating budget for HIV services at the facility, based upon actual resources identified during the assessment and the expected volume of services; and second, by using this budget to compute the percentage of total annual resources used for specific combinations of service and resource type.  For example, the RAM may tell us that 5% of total annual resources are used for personnel providing HTC services.  REMS uses this information to assign 5% of each Kwacha reaching the facility (in each calendar quarter) to “personnel” for HTC services.  We recognize that this approach creates some rigidity in the cost estimates.  The next version of REMS will allow for making RAM weights dynamic by linking to the output data, so that if there is an upsurge in testing, the 5% allocated to personnel for HTC would be adjusted upward (along with other resources within HTC) and shares allocated to other services would be decreased.***

 

- The paper implicitly makes the claim that efficiency data are very important for improving system performance (with which I agree, as a general point) and that the REMS system, in providing such data, is thus transforming health system efficiency. While this story is plausible, no evidence is presented that the interest expressed by the MoH in the REMS data are actually leading to transformational changes in health system performance. For example, the paper offers graphs of unit costs of certain types of care across different healthcare facilities showing highly heterogeneous costs per unit of service delivered. However, there is no indication of whether the large differences in cost of EMTCT are just a reflection of differences in maternal HIV prevalence – or whether there are clear differences in the efficiency of the delivery of the services provided. Thus, it is not clear from the paper that the estimation approach used will meaningfully result in better allocation of resources and improve efficiency.

**Response from the Authors:** ***While not shown in the graphs, there is an option to created stacked columns or sliced bars that would break down unit expenditure by the type of resource contributing to the results (i.e., staffing, supplies, equipment, etc.).  This allows for a comparison of how productively the fixed resources are being utilized, as these are what largely drive efficiency gains.  We think of REMS as a thermometer.  It can be used to document a fever, but it does not tell the user how to treat the fever.  The user is presented with this information to help target investigations of facility performance, identify outliers, perhaps identify data quality issues within the DHIS2 data, etc.  If corrective action is taken, REMS, like a thermometer, can be used to assess whether the patient is responding to the treatment, and if not, to catalyze additional investigations.  The goal of REMS is to put actionable information in the hands of persons who are asked to manage the health system, information that currently doesn’t exist.  Asking health systems to become more efficient users of scarce resources without providing tools to monitor resource use is unlikely to result in more efficient health systems.  All too often we see a standard resource package assigned to a facility based solely on its designation in the health system hierarchy, and not tied to the service volumes for which it is expected to deliver.***

 

- The paper makes several statements that argue that the process followed by FHI 360 was critically important to the success of the implementation. One such example: *Ensuring ownership by the Ministry of Health (MoH), Ministry of Finance (MoF) and Ministry of Community Development and Social Welfare (MCDSW) was critical to the project’s immediate success and long-run sustainability.*All of these statements are plausible, but there is no evidence provided to support the assertions. Would the implementation of a similar system in another country require the same degree of complexity in the implementation process? Is there evidence that the implementation would not have been successful if it had just been implemented by the MoH and subsequently revealed to the other Ministries? etc. As such, they appear to be statements of opinion by the authors that the approach they took was the correct/efficient/ethical approach that should be replicated if another country were to consider implementing such a system. It perhaps goes without saying that it is especially important to qualify such opinions when the authors are those who implemented the approach being assessed. A scientific article would normally make such statements only after having tested and validated the assertions using some scientific method and using observers who are as unbiased as possible.

**Response from the Authors:  *We acknowledge the concern about evidence to support these statements and have edited the text to make clear that these are statements of opinion.  In this specific example, access to the financial data is dependent upon the MoF, National and Provincial Level systems are run by the MoH and District Level systems are managed by MCDSW; with such inter-Ministerial complexity, it was critical that all three parties were on-board, engaged and in agreement about how REMS was to be built and accessed.  The deployment of a user-centered design process was partly in response to previous experiences where a solution was imposed from outside with little (if any) uptake or interest in supporting the system. We have expanded our discussion of this issue in the revised manuscript*** *.*

Thus, the paper in its current form is not a scientific publication but rather a well-written, well-organized report of the development and implementation of a health data system. It should not be published in the form of a scientific publication but rather as a report. Even as a report it should acknowledge (if true) the lack of rigorous validation of its estimation method and call for such validation. Such validation would not only give the Government of Zambia greater confidence in the estimates the system produces but provide much more useful information to other countries considering implementation of a similar system.

**Response from the Authors:** ***We agree.  Our award was funded to 1) develop a prototype, 2) show that the GRZ was willing to participate in the design and development of REMS in Zambia, and 3) implement the system in two Provinces.  As such what we have now is essentially a functioning proof of concept for which we are actively seeking further investments to take the system to the next level (expand geographically and into additional health program areas) as well as introduce internal feedback loops within the program logic and create a user-interface to generate performance reports and query the facility assessment data.  Our intent in publishing this Open Letter is to raise awareness of the work to date in hopes of securing funding for further validation and development of the system.***

In addition, the report should recognize that comparisons of unit costs without any assessment of the sources of the unit cost differences can provide seriously misleading information to decision-makers. If they are going to share examples of revealed heterogeneity being usefully addressed, it would be very helpful to show that the system can identify the source of the cost differences and that those have led to changes that have improved system performance. I often refer to justifiable and unjustifiable heterogeneity (which depends upon the perspective of the observer). As an example, within Zambia there is more than an order of magnitude of difference in HIV prevalence from one locality to another. It stands to reason that the unit cost of HIV services (especially prevention services) would therefore also (justifiably) vary greatly from one locality to another. Revealing (true) heterogeneity among localities can therefore represent differences in efficiency of service delivery (that needs to be addressed with improved management of service delivery) or differences in return to investment in prevention (which might be addressed by reallocating prevention services to localities where the returns are greater). At a minimum the report should temper the enthusiasm of the authors regarding the utility of the raw system output – or it should reveal that the system output is already far more nuanced than the current example suggests.

**Response from the Authors:*  As mentioned above, the current system is configured to allow users to examine sources of unit cost heterogeneity across facilities and over-time.  We are not claiming to have produced a perfect performance monitoring system, but rather to have laid the foundation for a more complete and robust system.  To our knowledge, no country in Sub-Saharan Africa (other than Zambia) has a public-sector enterprise cost accounting tool for health services currently in place.  We are hoping that REMS will enable more effective management of the health system in Zambia in a time when countries are being asked to take on a larger share of the financing of the health sector.  Our co-author from the MoH has stated that he believes that a fully built REMS system could achieve a 40% efficiency gain systemwide by reallocating resources to where they can be more productively utilized.  We look forward to using REMS system to validate this assertion, as REMS output is used to reallocate resources across facilities and reductions in the unit cost of care are documented over time.***

I would like to acknowledge the assistance of Madhav Nekkar in preparing this review. I accept full responsibility for its content.
